# Muscle Activity of the Latissimus Dorsi after Tendon Transfer in Patients with Rotator Cuff Tears

**DOI:** 10.3390/jcm9020433

**Published:** 2020-02-05

**Authors:** Pit Hetto, David Spranz, Felix Zeifang, Sebastian I. Wolf, Stefan van Drongelen, Michael W. Maier, Boris Sowa

**Affiliations:** 1 Clinic for Orthopedic and Trauma Surgery, Heidelberg University Hospital, 69118 Heidelberg, Germany; hettopit@gmail.com (P.H.); david.spranz@med.uni-heidelberg.de (D.S.); sebastian.wolf@med.uni-heidelberg.de (S.I.W.); m.w.maier@web.de (M.W.M.); 2 Clinic for Orthopedic Surgery, Ethianum, 69115 Heidelberg, Germany; felix.zeifang@med.uni-heidelberg.de; 3 Dr. Rolf M. Schwiete Research Unit for Osteoarthritis, Orthopaedic University Hospital Friedrichsheim gGmbH, 60528 Frankfurt/Main, Germany; Stefan.vanDrongelen@friedrichsheim.de

**Keywords:** latissimus dorsi tendon transfer, upper extremity, 3D motion analysis, surface EMG, activities of daily life, muscle activity, posterosuperior cuff tears

## Abstract

**Background**: Massive irreparable posterosuperior rotator cuff tears may result in the loss of external rotation. Most of the patients with posterosuperior rotator cuff tears suffer from a restriction in their daily life actions. Latissimus dorsi tendon transfer (LDTT) is a method to restore abduction and external rotation in these patients. However, the behavior of the LD after the transfer is not clear yet. Few studies have analyzed the activity of the LD after transfer. The goal of this study was to examine the function of the LD postoperatively in follow-up examinations during activities of daily life (ADLs) and during different movements evaluated by measuring the range of motion (ROM). **Methods**: We examined 12 patients 4.3 years (1–9 years) after LDTT, using simultaneous 3D motion analysis and surface Electromyography (sEMG) of 12 muscle parts; the opposite, nonaffected side was assessed as a control. The measurement protocol included two standardized movements (exorotation with an adducted arm and exorotation with 90° abduction) as well as two ADLs (combing hair and perineal care). **Results**: An average of 4.3 years (1–9 years) after LDTT, 9 of the 12 subjects showed EMG activity in the transferred LD during glenohumeral external rotation. During the endorotation phase, either little activity was registered or only the pectoralis major was active. During the ADLs, 6 out of 12 subjects showed EMG activity in the transferred LD while “combing hair”, whereas all subjects showed EMG activity during perineal care. **Conclusion**: The LD showed partial activity in its new role as an exorotator, but no clear difference was observed between some of the movements as well as in comparison with the healthy shoulder. Further studies will need to be conducted to better differentiate voluntary contractions and co-contractions as well as tension and extension in the muscle.

## 1. Introduction

Patients with an irreparable rotator cuff tear, especially posterosuperior cuff tear, may not only suffer pain but also have their daily life actions restricted. Movements requiring exorotation or elevation are limited the most. Therefore, muscle transfer is seen as a valid option for young patients who show no signs of arthritis in the shoulder joint, in order to optimize muscle function and to reduce pain [1]. Namdari et al. showed that patients with a cuff tear had positive outcomes after muscle transfer, with an improvement of pain as well as augmented ROM with a flexion of 137° (from 102° preoperative), exorotation of 27° (from 17° preoperative), and a constant score of 73.2 (45.9 preoperative) [2]. For the muscle transfer itself, multiple operations have been described, such as the transfer of the latissimus dorsi (LD) by Gerber et al. and Miniaci and MacLeod [3,4]. As an alternative, the transfer of the teres major was described by Celli et al. [5]. According to Gerber at al., the LD transfer is the most efficient—and most commonly used—technique to improve ROM [6]. In our hospital, the commonest technique for transferring the LD in patients with posterosuperior cuff tear is the method of Habermeyer and Herzberg, with a transfer of the LD to the greater tuberositas [7]. The LD transfer provides the advantages of muscle excursion and muscle tension but, for the transfer to work, it needs an intact subscapularis to restore an adequate muscle balance in the glenohumeral joint [1,3,8]. 

The behavior of the LD after the transfer is still not clear. Few studies analyzed the activity in the LD after the transfer, showing some activity during exorotation and flexion, especially in patients with good clinical outcomes. Interestingly, muscle activity was found in all patients during adduction [9]. Aoki et al. found activity in the LD which coincided with the activity of the supraspinatus during exorotation and abduction [10]. These studies analyzed muscle activity using EMG during isometric contractions. This brings up the question of whether, during functional movements in its new role, the muscle is active and thus functions as an agonist, and remains not active and, consequently, functions as an antagonist during movements of his old role. Ideally, the LD would only show activity during the new functions.

The goal of this study was to examine the LD activity postoperatively in follow-up examinations, during activities of daily life (ADL) and during measurements of the ROM while performing different movements. The control group comprised people with a healthy shoulder. Does the LD perform as an exorotator during ADLs and measurements of the ROM after an LD tendon transfer (LDTT)?

## 2. Materials and Methods

Twelve patients, with a mean age of 56.6 (±4.8 years), mean weight of 87.3 (±16.0 kg), and mean height of 1.75 (±0.12 m), underwent LDTT between October 2004 and July 2012. Of the 12 patients, 10 were men and 2 women. All the 12 patients were right-handed, 11 patients had surgery on the right side, and 1 patient had surgery on the left shoulder. Patients were examined after an average of 4.3 years (range 1–9 years) after the surgery. The non-operated shoulders underwent medical examination with anamnesis (previous surgery, pain, limitations) and testing (Jobe test, lift-off test, lag signs). If the “healthy” shoulders had previous surgery on the rotator cuff or biceps tendon, presented pain evaluated as >4 on the visual analogue scale (VAS), caused a limitation in daily life, or resulted positive in one or more tests, they were excluded. Of the 12 non-operated shoulders, 8 were considered as healthy shoulders and served as a control group. Subjects were informed about the study before giving written informed consent. The Medical Ethical Committee of the Heidelberg University Hospital approved the study (S-305/2007), and all patients consented to the study.

**Figure 1:**
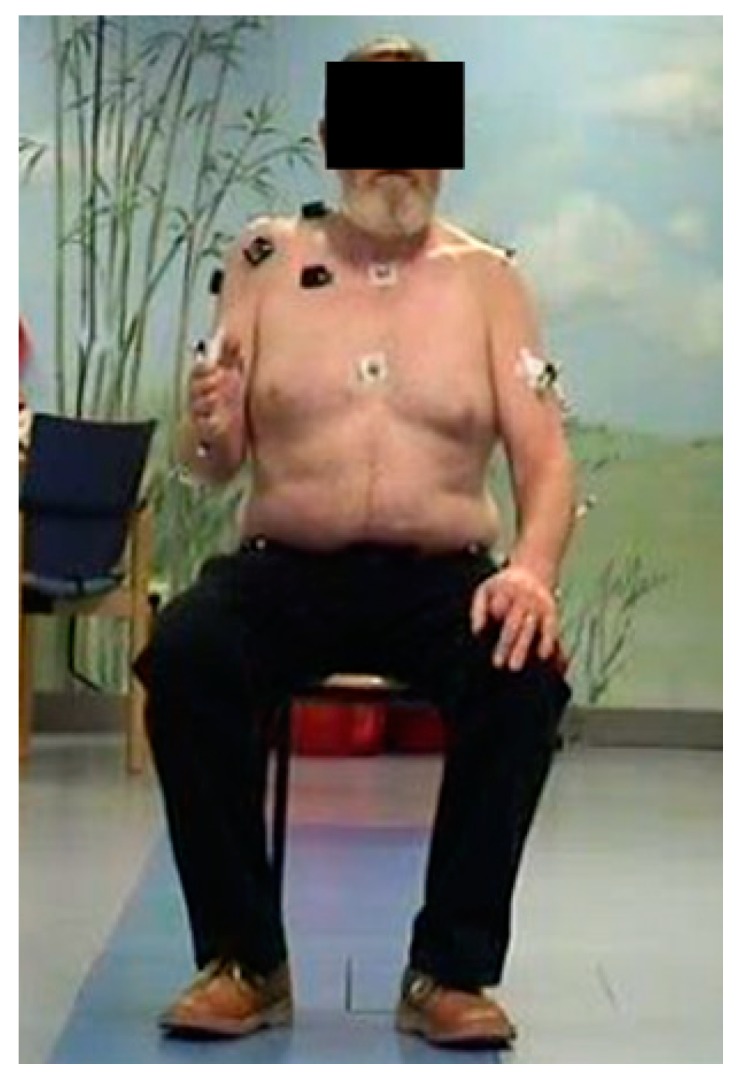
Overview of the measurement setup. A subject performing an exo/endorotation, with simultaneous collection of EMG and kinematics.

The 3D kinematic analysis was performed using a Vicon system (Vicon Motion System Inc., Lake Forrest, CA, USA), which consisted of 12 infrared video cameras with a resolution of 1.3 megapixels, recording the motions of the reflecting markers at a frequency of 120 Hz. The marker used in the study consisted of small spheres with a diameter of 8 mm, with an infrared reflective surface. We used single markers each consisting only of one sphere placed 2 mm from the skin and markers which consisted of two spheres linked by a 40 mm-long stick. The markers were placed on anatomic landmarks established by the Heidelberger Upper Extremity Model (HUX) [11,12]. The placement of the markers can be seen in Table 1 as well as Figure 1 and was performed according to Wu et al. [13]. The HUX model was validated in previous studies, with an intraclass correlation coefficients of 0.989 for intrasubject variability, 0.996 for intersubject variability, and 0.998 for intertester variability, in comparison to a manual goniometer model [11].

EMG of 12 muscle parts (deltoid (3), trapezius (3), pectoralis major, infraspinatus, teres major, latissimus dorsi, long head biceps, long head triceps) was recorded simultaneously with a bi-polar surface EMG equipment at 1080 Hz (Delsys TrignoTM, Delsys^®^, Natick, MA, USA). The skin was dry-shaved where needed and cleansed with alcohol pads before applying the electrodes, as described by Cram [14]. The averaged rectified EMG was used for analysis. Muscles were considered active if EMG was more than 2 times the resting EMG activity [15] Two standardized movements (exorotation with an adducted arm and exorotation with 90° abduction) as well as two ADL (combing hair and perineal care) were performed. 

The statistical analysis was performed using SPSS Version 16.0 (SPSS Inc., Chicago, IL, USA). Group mean values (MV) and standard deviations (SD) were calculated; *p* < 0.05 was considered significant.

## 3. Results

**Figure 2:**
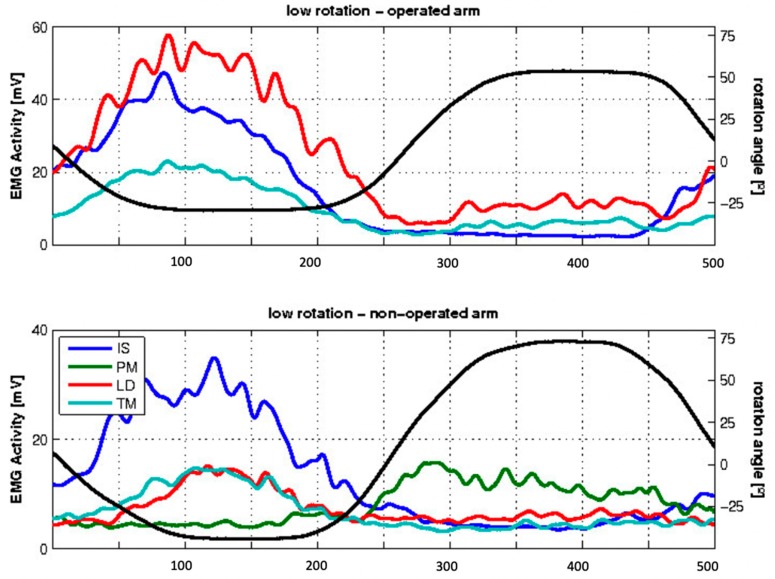
Mean EMG activity of the examined patients during low exorotation. The x-axis indicates the frames, and the y-axis indicates the EMG activity in mV on one the left, represented by colored lines corresponding to different muscles (infraspinatus (IS), pectoralis major (PM), latissimus dorsi (LD), and teres minor (TM).), and the angle of rotation on the right, represented by the black line. The negative values of the angle rotation correspond to the exorotation, and the positive values to the endorotation.

The 12 patients that were examined after 4.3 years had an average constant score of 62.9 (±14.6, range 35–86). All patients except one could perform the requested movements with the operated arm. Nine of the 12 subjects showed EMG activity in the transferred LD during glenohumeral external rotation, and 8 out of 8 subjects showed it in the non-operated side (Figure 2). Five patients could not externally rotate the arm further than the neutral position. Due to pathology of the contralateral arm, the measurements were only performed on the healthy arm in eight patients.

**Figure 3:**
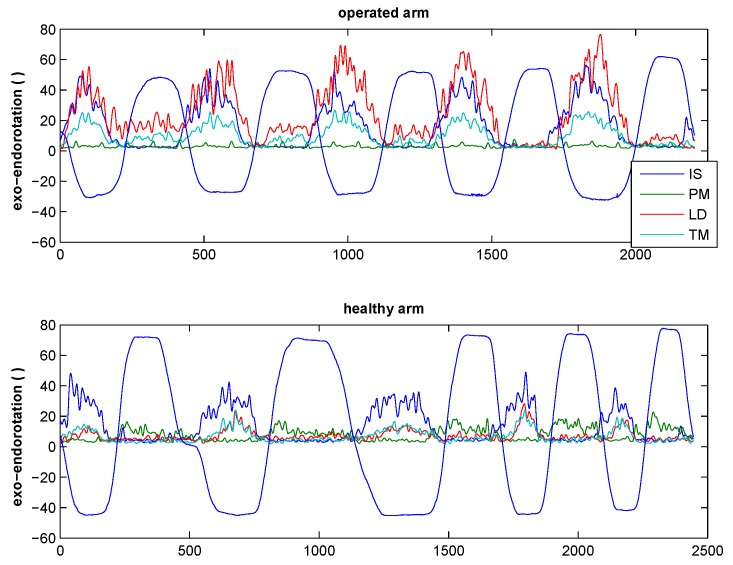
EMG and rotation angle over time for the patient showing more LD activity (red line) in the operated arm.

There was not a significant difference between the average EMG during full exorotation from the neutral position and that during full exorotation from the internally rotated position. For six patients, a comparison between the healthy and the operated arm was possible: two patients showed no difference, three showed lower EMG in the operated arm, and only one subject showed a higher EMG of the LD in the operated arm (Figure 3).

In most patients, the EMG showed co-contraction during the exorotation phase. Next to the infraspinatus, the teres major and the LD were active. During the endorotation phase, little activity was registered, or only the pectoralis major was active. This pattern was seen in both patients and healthy controls. 

**Figure 4:**
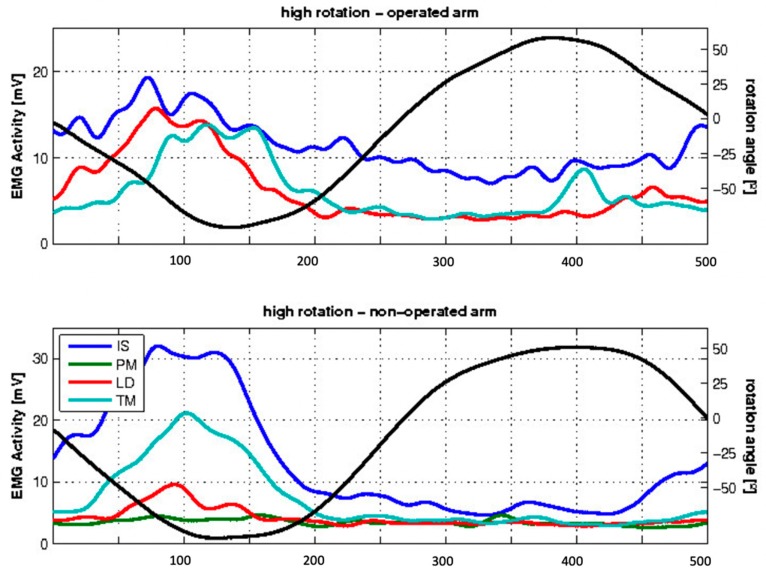
Mean EMG activity of the examined patients during high exorotation. The x-axis indicates the frames; the y-axis indicates, on the left side, the EMG activity in mV for the different muscles (IS, PM, LD, and TM), in colored lines and, on the right side, the angle of rotation, represented by the black line. Negative values show exorotation, and positive values endorotation.

During glenohumeral rotation in 90° of abduction, 10 out of 12 subjects showed EMG activity in the transferred LD during external rotation, whereas 2 out of 8 subjects showed this activity in the non-operated side (Figure 4). 

**Figure 5:**
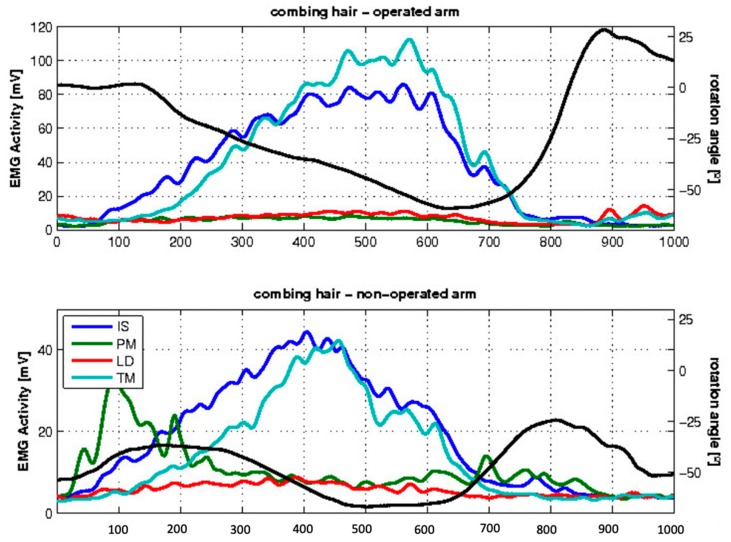
Mean EMG activity during the activity of daily life (ADL) of combing hair for the examined patients. The x-axis indicates the frames, and the y-axis indicates, on the left side, the EMG activity in mV of the different muscles (colored lines) and, on the right side, the angle of rotation represented by the black line. Negative values show exo-rotation, and positive values endorotation.

During the ADL of combing hair (large external rotation component), 6 out of 12 subjects showed EMG activity in the transferred LD during external rotation, whereas 3 out of 8 subjects showed EMG activity in the non-operated side (Figure 5).

**Figure 6:**
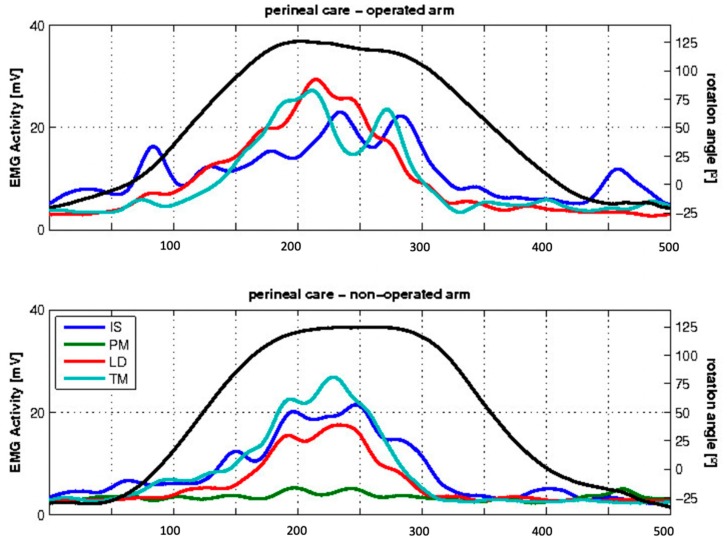
Mean EMG activity of the examined patients during the ADL of perineal care. The x-axis indicates the frames, and the y-axis indicates, on the left side, the EMG activity in mV of the different muscles (colored lines) and, on the right side, the angle of rotation represented by the black line. Negative values show exo-rotation, and positive values endorotation.

During perineal care, all subjects showed EMG activity in the non-operated and transferred LD during internal rotation (Figure 6). 

## 4. Discussion

The LD transfer has proved to be a valid option to restore both function and range of motion in patients with posterosuperior rotator cuff tear. Most of the previous studies have focused on assessing the range of motion of LD after transfer. The purpose of this study was to evaluate the activity of the LD after transfer during different movements with an exorotational component, using surface EMG.

At the mean postoperative follow-up of 4.3 years, activity in the LD for exorotation was detected in the operated arm as well as in the nonoperated side when the arm was kept at the side. It was difficult to distinguish if the function of the LD during this movement was, in fact, due to exorotation or to adduction to keep the arm at the side. During high exorotation, with the arm at 90° abduction, the transferred LD was active as an external rotator and/or as a stabilizer of the glenohumeral joint, allowing the deltoid muscle to contribute to shoulder motion more effectively. During the ADL of combing hair the LD probably functioned as an exorotator in some patients, although it showed activity in some healthy arms too. During perineal care, the transferred LD showed activity during its original function as an internal rotator. It was possible that the measured activity derived from tension or extension of the muscle, although the differentiation was difficult.

The LD showed EMG activity during exorotation; however, it was not possible to distinguish action and co-contraction of the transferred muscle.

Aoki et al. found a similar activity in the transferred LD. In most of their patients, the LD was active during exorotation, with a synergistic behavior to the supraspinatus. In some patients, the LD showed activity during its original functions of adduction and internal rotation [10]. However, this study was performed without 3D motion analysis; therefore, the EMG-recorded activity was not as precisely attributable to the motion and the position of the arm. Furthermore, no analysis of ADL was performed. 

Irlenbusch et al. performed an EMG analysis of the LD after transfer, over different intervals. It was found that the LD was mostly active when performing its new functions, especially during flexion and abduction, and less active during internal rotation. The activity of the LD during exorotation was not described. They attributed the functional improvement of the shoulder to active muscle contraction [16].

Similar results were found by Henseler et al. in their prospective trial. They reported an increased activity of the LD during abduction, whereas, preoperatively, it was mainly active during adduction. The activity was only measured during directional isometric movement of abduction or adduction [15]. 

Ippolito et al. performed a 3D motion analysis with surface EMG. To normalize the EMG activity during the trial runs and evaluate the EMG results, they used a different method, i.e., the measurement of the maximal voluntary isometric contractions (MVIC). They analyzed different movements but did not ADLs. They found, in contrast to other studies, that transfer of the LD did not change its overall functions; therefore, the LD mostly continued to function as an internal rotator [9].

Like the other published studies, our study does not show conclusive evidence for the activity and role of the LD after transfer. The reported studies showed very different results concerning the activity of the LD. The use of different methodologies in those studies makes a simple comparison difficult. Nonetheless, it remains clear that the new function of the LD has not been identified yet. Also, it is still uncertain if the LD transfer helps patients by providing the LD with a new function or if the beneficial effects of the operation are only due to the tenodesis.

The limitations of this study are the small number of patients and the fact that the study lacks preoperative data, as it was performed only postoperatively. The number of movements and ADLs are also limited.

For better results, the patients would need to be evaluated prospectively, with a longer follow-up, to evaluate the function of the LD. Furthermore, other movements and more ADLs should be analyzed, including activities which require more force than just that to bear the weight of the arm. The analyzed movements, with the low and high rotation, might have been unusual for this patient’s group, therefore provoking a co-contraction to stabilize the arm and the glenohumeral joint. The analysis of the response to ADLs might show how well a transferred LD has adopted its new functions as an exorotator; however, kinematics should be analyzed, as patients tend to use evasive movements to complete the task at hand if proper execution is not possible. Furthermore, the force applied during the movements should be evaluated.

## 5. Conclusions

The LD partially showed activity in its new role as an exorotator, but no clear difference was observed between some movements as well as in comparison with the corresponding healthy shoulder. Further studies are needed in order to better differentiate voluntary contractions and co-contractions as well as tension and extension in the muscle. 

## Figures and Tables

**Table 1:** Description of the placement of the markers.

Single Marker	Position
CLAV	Incisura jugularis of the sternum
STRN	Processus xiphoideus of the sternum
C7	Processus spinosus C7
T10	Processus spinosus TH10
RASI	Right spina illiaca anterior superior
LASI	Left spina illiaca anterior superior
RSHO	Middle of the lateral edge of the right acromion
LSHO	Middle of the lateral edge of the left acromion
RHUMS	Tuberositas deltoidea of the right humerus
LHUMS	Tuberositas deltoidea of the left humerus
RRAD	Processus styloideus radii of the right radius
LRAD	Processus styloideus radii of the left radius
RULN	Processus styloideus ulnae of the right ulna
LULN	Processus styloideus ulnae of the left ulna
Double marker	
RELB/RELBW	2 cm distal to the right olecranon
LELB/LELBW	2 cm distal to the left olecranon
